# Suppression of Proteoglycan-Induced Autoimmune Arthritis by Myeloid-Derived Suppressor Cells Generated *In Vitro* from Murine Bone Marrow

**DOI:** 10.1371/journal.pone.0111815

**Published:** 2014-11-04

**Authors:** Júlia Kurkó, András Vida, Tímea Ocskó, Beata Tryniszewska, Tibor A. Rauch, Tibor T. Glant, Zoltán Szekanecz, Katalin Mikecz

**Affiliations:** 1 Section of Molecular Medicine, Department of Orthopedic Surgery, Rush University Medical Center, Chicago, Illinois, United States of America; 2 Department of Rheumatology, University of Debrecen, Faculty of Medicine, Debrecen, Hungary; University Hospital Jena, Germany

## Abstract

**Background:**

Myeloid-derived suppressor cells (MDSCs) are innate immune cells capable of suppressing T-cell responses. We previously reported the presence of MDSCs with a granulocytic phenotype in the synovial fluid (SF) of mice with proteoglycan (PG)-induced arthritis (PGIA), a T cell-dependent autoimmune model of rheumatoid arthritis (RA). However, the limited amount of SF-MDSCs precluded investigations into their therapeutic potential. The goals of this study were to develop an in vitro method for generating MDSCs similar to those found in SF and to reveal the therapeutic effect of such cells in PGIA.

**Methods:**

Murine bone marrow (BM) cells were cultured for 3 days in the presence of granulocyte macrophage colony-stimulating factor (GM-CSF), interleukin-6 (IL-6), and granulocyte colony-stimulating factor (G-CSF). The phenotype of cultured cells was analyzed using flow cytometry, microscopy, and biochemical methods. The suppressor activity of BM-MDSCs was tested upon co-culture with activated T cells. To investigate the therapeutic potential of BM-MDSCs, the cells were injected into SCID mice at the early stage of adoptively transferred PGIA, and their effects on the clinical course of arthritis and PG-specific immune responses were determined.

**Results:**

BM cells cultured in the presence of GM-CSF, IL-6, and G-CSF became enriched in MDSC-like cells that showed greater phenotypic heterogeneity than MDSCs present in SF. BM-MDSCs profoundly inhibited both antigen-specific and polyclonal T-cell proliferation primarily via production of nitric oxide. Injection of BM-MDSCs into mice with PGIA ameliorated arthritis and reduced PG-specific T-cell responses and serum antibody levels.

**Conclusions:**

Our in vitro enrichment strategy provides a SF-like, but controlled microenvironment for converting BM myeloid precursors into MDSCs that potently suppress both T-cell responses and the progression of arthritis in a mouse model of RA. Our results also suggest that enrichment of BM in MDSCs could improve the therapeutic efficacy of BM transplantation in RA.

## Introduction

Rheumatoid arthritis (RA) is a chronic autoimmune inflammatory disease that leads to painful joint destruction and disability [1], [2]. Despite novel treatment strategies, not all patients respond to therapy. Although cell-based therapy such as transplantation of autologous bone marrow (BM) or hematopoietic stem cells is a promising option in both refractory RA [3] and therapy-resistant juvenile idiopathic arthritis [4], clinical remission in transplant recipients is still incomplete. Exploration of novel therapeutic options is needed in order to control immune responses that drive inflammation in these cases.

Research in recent years has uncovered a heterogeneous population of immature myeloid cells, called myeloid-derived suppressor cells (MDSCs). MDSCs with immunosuppressive capacity were initially described in tumor-bearing mice [5]. Although the vast majority of data comes from cancer research (reviewed in [6], [7]), accumulating evidence supports the role of MDSCs in chronic inflammatory and autoimmune disorders. A common feature of these pathological conditions is the release of a broad array of inflammatory mediators (growth factors and cytokines) that not only exert their effects on the affected organs, but also disturb myelopoiesis in the BM. While some of these mediators promote the expansion of MDSCs through stimulation of myelopoiesis, others inhibit full differentiation of myeloid precursors, thus contributing to the accumulation of MDSCs around malignant tumors or at sites of inflammation (reviewed in [8]). As the microenvironment under different pathological conditions varies, the phenotypic and the functional properties of MDSCs can be diverse [9], [10]. MDSCs do not constitute a homogenous cell population, rather, they are a mixture of “immature” forms of monocytes and granulocytes. What classifies them as an integrated system is their shared ability to suppress adaptive immune responses [8], [10].

MDSCs in mice express the common myeloid markers CD11b (α chain of α_M_β_2_ integrin, an adhesion molecule present on monocytes and granulocytes) [11] and Gr-1 [8], [12]. The epitope of the widely used anti-Gr-1 monoclonal antibody (mAb, clone RB6-8C5) is present on two molecules, Ly6G and Ly6C, which are encoded by separate genes and expressed in granulocytic and monocytic cells, respectively. Based on cell surface staining with mAbs against CD11b, Gr-1, Ly6G, and Ly6C, the following subtypes of murine MDSCs have been identified: CD11b^+^Gr-1^+^Ly6G^hi^Ly6C^lo^ granulocytic, and CD11b^+^Gr-1^+^Ly6G^−^Ly6C^hi^ monocytic MDSCs [12], [13]. These two subsets also display distinct cellular morphology and may employ different strategies to suppress immune responses in malignant, infectious, and autoimmune disease models [13]–[15]. The ultimate in vitro tools for identifying MDSCs are functional assays testing the ability of “MDSC-like” cells to suppress T-cell responses [8].

Although MDSCs in cancer patients inhibit anti-tumor immunity and thus promote tumor progression [6], [16], the immunosuppressive ability of MDSCs could be exploited to limit further tissue damage in disorders like RA, where the pathological mechanism revolves around the excessive activation of the adaptive immune system [9]. This statement is corroborated by several recently published studies involving successful adoptive cell transfer of MDSCs in animal models of inflammatory bowel disease [17], autoimmune uveoretinitis [18], type I diabetes [19], multiple sclerosis (MS) [14] and collagen-induced arthritis (CIA) [20]. All these studies depended on one key factor: a good source of MDSCs.

In a previous study [21] we reported that synovial fluid (SF) in the joints of mice with proteoglycan (PG)-induced arthritis (PGIA) [22], [23], an autoimmune animal model of RA, contained a cell population with a granulocytic phenotype and a biological activity resembling MDSCs. Upon co-culture of SF cells with T cells in the presence of antigen (Ag)-loaded dendritic cells (DCs), T-cell proliferation was profoundly inhibited, thereby confirming the suppressor activity of SF-MDSCs. Experiments employing inhibitors of MDSC products revealed that these cells exerted their suppressive effect via nitric oxide (NO) and reactive oxygen species (ROS) production [21]. SF-MDSCs also significantly inhibited the maturation of DCs through down-regulation of the major histocompatibility class II (MHC II) molecule and the co-stimulatory molecule CD86, resulting in impaired Ag presentation by the affected DCs. Phenotypic characterization revealed that the SF cell population was dominated by CD11b^+^Gr-1^+^ Ly6G^hi^Ly6C^int/lo^ (granulocyte-like) MDSCs, but cells with CD11b^+^Gr-1^+^Ly6G^−^Ly6C^hi^ (monocytic) phenotype were also detectable [21]. Interestingly, unlike in a recently published study [20] where CD11b^+^Gr-1^+^ cells isolated from the spleens of mice with CIA had suppressor activity toward T cells, splenic CD11b^+^Gr-1^+^ cells from mice with PGIA did not suppress T-cell proliferation in vitro, only SF cells did [21]. This led us to the conclusion that the inflammatory microenvironment (e.g., locally produced cytokines and growth factors) within the affected joints has the utmost importance in not only promoting the recruitment of granulocytic precursors, but also keeping these cells in an immature state and endowing them with immune modulatory properties.

Our data suggested that SF-MDSCs could be exploited for therapeutic purposes to prevent the expansion of pathogenic T cells in vivo. However, the amount of SF that could be harvested from the small mouse joints was a serious limiting factor for cell transfer-based therapeutic studies, which prompted us to explore alternative sources of MDSCs. We found that murine BM was an excellent source of MDSCs and their precursors that could be expanded in culture in an appropriate cytokine milieu. We chose in vitro enrichment instead of antibody (Ab)-based positive selection of BM-MDSCs because Ab binding to either CD11b or Gr-1 on the cell surface has been shown by us and others to impair the trafficking, survival, and suppressive function of myeloid cells [11], [24], [25]. In the present study, we report on the development of a method for generating large amounts of MDSCs from BM (BM-MDSCs) whose characteristics are partially similar to those found in the SF of mice with PGIA, but are more powerful than SF-MDSCs in suppressing the Ag-independent, polyclonal proliferation of T cells. We have also found that BM-MDSCs are able to inhibit the progression of adoptively transferred PGIA following injection of these cells into mice with early arthritis symptoms.

## Materials and Methods

### Mice, immunization, and assessment of arthritis

Adult female BALB/c mice were obtained from the National Cancer Institute (Frederick, MD). Enhanced green fluorescent protein-lysozyme M transgenic (EGFP-LysM-Tg) mice [26] were back-crossed to the BALB/c background for 10 generations [21], [27]. Spleens of naïve PG-specific T cell receptor transgenic (PG-TCR-Tg) BALB/c mice (recognizing a dominant epitope within the G1 domain of human PG [28]) were used as a source of PG/G1-specific T cells. BALB/c mice with the severe combined immunodeficiency (scid) mutation (SCID mice) [27], [29] were purchased from the National Cancer Institute.

To induce arthritis, adult female wild type (wt) BALB/c mice were injected intraperitoneally (i.p.) with 100 µg of human PG protein [22] emulsified in dimethyl-dioctadecyl-ammonium bromide (Sigma-Aldrich, St Louis, MO) adjuvant in sterile phosphate buffered saline (PBS) 3 times 3 weeks apart [23], [30]. PG was extracted from human cartilage as described previously [21]–[23]. Cartilage was donated by patients undergoing joint replacement surgery. Written informed consent was obtained from each patient. Collection of surgical specimens was approved by the Institutional Review Board of Rush University Medical Center (Chicago, IL). After the second injection of human PG, the limbs of immunized mice were examined for signs of arthritis. A standard visual scoring system (based on swelling and redness, ranging from 0 to 4 for each paw, 0–16 per mouse) was used for the assessment of disease severity. All experiments involving animals were conducted in accordance with the recommendations of the Guide for the Care and Use of Laboratory Animals of the National Institutes of Health. The animal studies were approved by the Institutional Animal Care and Use Committee of Rush University Medical Center (Permit Number: 11–046).

### Collection of serum and cells/organs from mice, and histology

Blood for cell analysis and measurement of anti-PG Ab titers was drawn from mice under deep anesthesia induced by intramuscular injection of a Ketamine-Xylazine cocktail. Mice were then euthanized by carbon dioxide inhalation, and spleen, BM, joint-draining lymph nodes (LNs) were collected. SF was harvested in heparin containing tubes from arthritic ankles, knees, and forepaws at the peak of the disease (inflammation score: 8–16 per mouse) after puncturing and gently pressing of the joints. Red blood cells were eliminated by hypotonic lysis and single cell suspensions were prepared from the harvested tissues and fluids.

For histology, hind limbs were dissected, fixed with formalin, decalcified, and embedded in paraffin. Sagittal sections, cut from the paraffin-embedded tissue, were stained with hematoxylin and eosin and examined under a Nikon Microphot light microscope (Nikon, Melville, NY). Microphotographs of the ankle (tibio-talar) joints were prepared using a digital color CCD camera (Coolsnap; RS Photometrics, Tucson, AR) and MetaMorph software (Molecular Devices, Sunnyvale, CA).

### Generation of MDSCs and DCs from BM

MDSCs were generated from BM of naïve wt or EGFP-LysM-Tg BALB/c mice. Femurs and tibias were collected under aseptic condition, and BM was flushed out with sterile PBS. After red blood cell lysis, BM cells were counted (the number of cells was usually 3–4×10^7^ per mouse), and seeded in Petri dishes at a density of 5×10^5^ cells per ml of Dulbecco’s Modified Eagle Medium (DMEM; Sigma-Aldrich) containing 10% fetal bovine serum (FBS) (Hyclone, Logan, UT). In preliminary dose-finding experiments the BM cells were cultured for 3 to 7 days in the presence of varying doses of recombinant murine granulocyte macrophage colony stimulating factor (rmGM-CSF; Peprotech, Rocky Hill, NJ) and recombinant murine interleukin-6 (rmIL-6; Peprotech), or with a combination of rmGM-CSF, rmIL-6, and recombinant murine granulocyte colony stimulating factor (rmG-CSF; Peprotech). On the basis of phenotypic and functional characteristics, the optimal protocol for BM-MDSC generation was found to be a 3-day culture of BM cells in the presence of rmGM-CSF, rmIL-6, and rmG-CSF (10 ng/ml each).

DCs, as Ag-presenting cells (APCs), were also generated from BM of naïve wt BALB/c mice by culturing BM cells for 9 days in the presence of 40 ng/ml rmGM-CSF, as described previously [21], [31].

### Phenotypic analysis of cells by flow cytometry

To assess the effect of BM-MDSCs on DC maturation, DCs were cultured alone or in the presence of BM-MDSCs for 2–3 days prior to flow cytometric measurement of the levels of MHC II and CD86 expression. Similarly, T cells were co-cultured with Ag-loaded DCs in the absence or presence of BM-MDSCs (as described below for T-cell proliferation assays), and the effect of BM-MDSCs on regulatory T cell (Treg) differentiation and cytokine production was determined by flow cytometry after intracellular staining (see below).

After harvesting the cells of interest, cells were suspended in flow staining/washing buffer (PBS containing 0.05% bovine serum albumin and 0.05% sodium azide). Prior to surface staining with fluorochrome-tagged mAbs, Fc receptors were blocked with purified anti-CD16/CD32 mAb (Fc block; rat mAb, clone 2.4G2; BD Biosciences, San Diego, CA) for 10 minutes at 4°C. Immunostaining was performed using fluorochrome-conjugated mAbs (obtained from BD Biosciences, eBioscience, or BioLegend, San Diego, CA) against the following cell surface markers: CD11b (rat mAb, clone M1/70), Gr-1 (rat mAb, clone RB6-8C5), Ly6C (rat mAb, clone HK1.4), Ly6G (rat mAb, clone 1A8), F4/80 (rat mAb, clone RM8), CD115 (rat mAb, clone AFS98), CD80 (hamster mAb, clone 16-10A1), CD11c (hamster mAb, clone N418), MHC II (I-A^d^/I-E^d^) (rat mAb, clone M5/114.15.2), CD86 (rat mAb, clone GL-1), CD3 (hamster mAb, clone 145-2C11), CD4 (rat mAb, clone GK1.5), and CD25 (rat mAb, clone PC61). Separate cell aliquots were stained with fluorochrome-labeled isotype-matched rat or hamster control IgGs. For detection of Tregs, cells were first stained for CD4 and CD25, permeabilized, and stained for intracellular FoxP3 using a mouse FoxP3 staining kit (Cat. No. 8-8111-40; eBioscience). A staining protocol and a fixation/permeabilization kit (Cytofix/Cytoperm kit with GolgiStop from BD Biosciences) were employed to detect intracellular cytokines. In brief, the cells (2×10^6^/ml culture medium) were first incubated with 10 ng/ml phorbol-13-myristate acetate (PMA, Sigma), 1 µg/ml Ionomycin (Invitrogen, Grand Island, NY), and 1 µl/ml GolgiStop (2 µM Monensin) for 4 hours. After surface staining for CD4 (rat mAb, clone GK1.5) the cells were fixed, permeabilized, and stained with fluorochrome-conjugated mAb to murine interferon gamma (IFNγ) (rat mAb, clone XMG1.2; BioLegend) or IL-10 (rat mAb, clone JES5-16E3; eBioscience). Flow cytometry was performed using a BD FACS Canto II instrument, and data were analyzed with FACS Diva software (BD Flow Cytometry Systems, San Jose, CA).

### Immunofluorescence imaging and cytospin preparations

Occasionally, BM-MDSCs, generated from EGFP-LysM-Tg mice (which express EGFP only in cells of myeloid origin) [31] were used for fluorescence imaging. In brief, after BM-MDSCs were immunostained with fluorochrome-labeled mAbs to Ly6G and Ly6C (specified above), a small aliquot of cell suspension was placed in a 0.5 mm-deep imaging chamber (Invitrogen). The cells were visualized using a Prairie Ultima two-photon microscope system (Prairie Technologies, Middleton, WI), and images were created with Imaris software (Bitplane, South Windsor, CT) as described previously [27].

For analysis of cell morphology, BM-MDSCs or SF cells were spun onto glass slides, air dried, and stained with Wright-Giemsa solution (Sigma-Aldrich). Cytospin preparations were viewed and photographed as described for joint histology.

### Measurement of GM-CSF, IL-6, and G-CSF levels in mouse serum and SF

Concentrations of GM-CSF, IL-6, and G-CSF in serum and cell-free SF samples of arthritic mice were measured using sandwich enzyme-linked immunosorbent assay (ELISA) kits from Peprotech (Cat. No. 900-M55, 900-M50, and 900-K103, respectively). Serially diluted (1∶50–1∶400) serum and SF samples and the appropriate standards were incubated in plates coated with anti-GM-CSF, anti-IL-6, or anti-G-CSF Abs, and plate-bound material was detected according to the manufacturer’s instructions. Absorbance at 450 nm was read by a Synergy 2 ELISA reader (BioTek Instruments, Winooski, VT).

### Purification of T cells and depletion of Ly6C^hi^ monocytic MDSCs

T cells were purified from the spleens of naive PG-TCR-Tg BALB/c mice by negative selection using an EasySep Mouse T Cell Enrichment Kit (Cat. No. 19751; StemCell Technologies, Vancouver, BC, Canada). The purity of T cells, verified by flow cytometry, was greater than 95% in all cases.

Depletion of Ly6C^hi^ (monocytic) cells from the total BM-MDSC population was carried out using an EasySep Biotin Selection Kit (StemCell Technologies). Unwanted cells were targeted with a biotinylated mAb against Ly6C (rat mAb, clone HK1.4), followed by immunomagnetic depletion of the mAb-tagged cells. This resulted in the removal of essentially all Ly6C^hi^ (but not Ly6C^int/lo^Ly6G^hi^) BM-MDSCs, as confirmed by flow cytometry.

### Assays for determining MDSC-mediated suppression of T-cell proliferation

For assessment of suppression of Ag-dependent T-cell proliferation, first the DCs were cultured overnight with the recombinant G1 domain of human PG (rhG1; 7.5 µg/ml) [32] as Ag in the absence or presence of BM-MDSCs, Ly6C^hi^ cell-depleted BM-MDSCs, or SF cells (as suppressors) in quadruplicate wells of 96-well plates. T cells purified from the spleens of naive PG-TCR-Tg mice were added and co-cultured for 5 days at a T cell:DC:suppressor cell ratio of 1∶0.3∶1. Background controls included the following: T cells and DCs co-cultured without Ag (rhG1) and each suppressor population cultured alone for the same length of time (5 days). The cells were pulsed with [^3^H]thymidine (Perkin Elmer, Waltham, MA) at 1 µCi/well for the last 18 hours of culture, and isotope incorporation (counts per minute: cpm) was measured in a MicroBeta scincillation counter (Perkin Elmer).

To assess Ag-independent suppression of T-cell proliferation, 96-well plates were first coated with purified mAbs against CD3 (hamster mAb, clone 145-2C11) and CD28 (hamster mAb, clone 37.51) (1 µg of each per well in 100 µl sterile sodium carbonate buffer, pH 9.6). T cells were added to the coated wells alone, or with an equal number of BM-MDSCs, Ly6C^hi^ cell-depleted BM-MDSCs, or SF cells as suppressors. Background controls were T cells cultured in uncoated wells and suppressors cultured in anti-CD3/CD28-coated wells. T-cell proliferation was measured on day 4 of culture as described above.

In all cases, the results of proliferation assays were expressed as percent suppression [15] (after correction for backround proliferation) using the following equation:

% suppression = 100– [(cpm with suppressors/cpm without suppressors) ×100].

To inhibit MDSC-mediated suppression, the following inhibitors of MDSC products [21] were added to the co-cultures of T cells, Ag-loaded DCs, and BM-MDSCs (or anti-CD3/CD28-stimulated T cells and BM-MDSCs): N^ω^-hydroxy-nor-arginine (nor-NOHA; 0.5 mM), an inhibitor of arginase 1; N^G^-monomethyl-L-arginine acetate (L-NMMA; 0.5 mM) and 1400W (0.1mM), inhibitors of inducible nitric oxide synthase (iNOS); Z-VAD-FMK (0.1 mM), an inhibitor of caspases and caspase-mediated apoptosis (all inhibitors were purchased from Calbiochem, Gibbstown, NJ); or the ROS scavenger catalase (1,000 U/ml, Sigma-Aldrich). Cell proliferation results were expressed as % suppression in the presence and absence of each inhibitor.

### Reverse transcription-polymerase chain reaction (RT-PCR)

As described in our previous study [21], the transcript for murine iNOS (*Nos2*) was expressed at much lower levels in spleen cells than SF cells obtained from arthritic mice. Therefore, we used spleen cells as a reference control to determine if the *Nos2* gene was also upregulated in BM-MDSCs. Total RNA was isolated from BM-MDSCs and control spleen cells using TRI reagent (Sigma-Aldrich) according to the manufacturer’s instructions. cDNA was synthesized employing a SuperScript First Strand kit (Invitrogen), and PCR was performed using HotStart Taq Plus enzyme (Qiagen, Carlsbad, CA) in 35 cycles (95°C for 20 sec, 57°C for 30 sec, and 72°C for 45 sec) with a final extension at 72°C for 10 min in a C1000 Thermal Cycler (Bio-Rad, Hercules, CA). A murine *Nos2*-specific primer pair (*Nos2* forward 5′-CCCTTCCGAAGTTTCTGGCAGCAGC-3′, and *Nos2* reverse 5′-GGCTGTCAGAGCCTCGTGGCTTTGG-3′) was used to detect the *Nos2* transcript, and an *Actb* gene-specific primer pair (*Actb* forward 5′-TGGCTCCTAGCACCATGAAGATCA-3′ and *Actb* reverse 5′-ATCGTACTCCTGCTTGCTGATCCA-3′) served for detection of the housekeeping gene encoding β-actin. After amplification, samples were loaded onto 1.5% agarose gels.

### Western blot

BM-MDSCs and control spleen cells were lysed in cold RIPA buffer containing a Halt protease inhibitor mixture (Pierce/Thermo Fisher, Rockford, IL), and the protein content was determined using the bichinconic acid assay (Pierce). Proteins from cell lysates (20 µg protein each) were loaded onto and resolved in 7.5% SDS-PAGE gels (Bio-Rad) under reducing conditions. The proteins were then transferred to nitrocellulose membranes. The membranes were blotted with an anti-mouse iNOS mAb (mouse mAb, Cat. No. sc-7271; Santa Cruz Biotechnology, Dallas, TX) at a 1∶500 dilution. Horseradish peroxidase (HRP)-conjugated rabbit anti-mouse IgG1 (Invitrogen) was used as a secondary Ab at a 1∶10,000 dilution. The protein bands were visualized using the enhanced chemiluminescence method (Amersham/GE Healthcare Life Sciences, Piscataway, NJ). The membranes were stripped and re-probed with a HRP-conjugated mAb to β-actin (mouse mAb, clone mAbcam 8226; Abcam, Cambridge, MA) at a 1∶5,000 dilution to ensure equal sample loading.

### Measurement of iNOS activity

To measure iNOS enzymatic activity (NO production) in the supernatants of 2-day co-cultures of murine BM-MDSCs, DCs and T cells, a nitrite/nitrate colorimetric assay was performed according to the manufacturer’s protocol (Cayman Chemical, Ann Arbor, MI). Supernatants of spleen cell cultures, containing the same number of cells as the co-cultures, were used as a reference. Samples were run on a BioTek microplate reader and absorbance was measured at 540 nm. A standard curve was generated using nitrate standards serially diluted between 5 µM and 35 µM. Results were expressed as total nitrate concentration (µM).

### Induction of adoptively transferred PGIA in SCID mice and BM-MDSC transfer

Adoptive cell transfer from wt BALB/c to SCID BALB/c mice is an ideal tool for investigating the in vivo distribution and effects of donor cells, as the syngeneic SCID mice exhibit complete tolerance to the wt donor cells, allowing these cells (e.g., lymphocytes) to expand rapidly in vivo [33]. SCID BALB/c mice also develop PGIA after spleen cell transfer from arthritic donors in a more uniform and synchronous manner than wt BALB/c mice following PG immunization [27], [33] . To induce adoptively transferred PGIA in SCID BALB/c mice, spleen cells from arthritic wt BALB/c donors were injected intravenously into SCID recipients (∼10^7^ cells/mouse). At the time of spleen cell transfer, SCID mice also received 100 µg of human PG (without adjuvant) i.p. to re-activate the donor cells in vivo [27], [33]. When arthritis started to develop (day 15 after the first splenocyte transfer), mice were divided into two groups (n = 10 mice/group) with mean disease scores of 2.0 and 2.05, respectively. One group received a second transfer of 10^7^ splenocytes with 100 µg of human PG i.p., and the other group was co-injected i.p. with spleen cells and BM-MDSCs (∼10^7^ of each cell type/mouse) together with the same dose of PG. Control mice (injected with only splenocytes and PG twice) and BM-MDSC-treated mice (also receiving BM-MDSCs with the second injection of spleen cells and PG) were examined twice a week for disease severity and scored as described for the primary form of PGIA. Mice were sacrificed on day 34 after the first cell transfer for determination of joint histopathology and PG-specific immune responses. Arthritis severity data were collected from 2 independent experiments, each having 5 mice per group (10 mice total per group).

We also carried out a separate experiment on a limited number of SCID mice (n = 3) to assess the distribution of transferred BM-MDSCs in various tissues. In order to distinguish the transferred cells from the recipients’ own MDSCs, BM-MDSCs were generated from EGFP-LysM-Tg BALB/c mice that express EGFP in myeloid cells [27], [31]. As described above, SCID mice injected with PG and 10^7^ spleen cells from arthritic wt BALB/c mice on days 0 and 15 also received 5×10^6^ EGFP^+^BM-MDSCs i.p. on day 15. This amount of BM-MDSCs (half of the therapeutic dose) only weakly inhibited arthritis progression, which enabled us to detect donor cells in the SF of recipient mice. On day 34, blood, SF, BM, spleen, and joint-draining LNs were harvested from the recipient mice. The cells were immunostained for CD11b, Ly6C, and Ly6G, and the subset composition of EGFP^+^CD11b^+^ donor cells was determined by flow cytometry.

### Measurement of PG-specific T-cell responses and serum Abs in SCID mice

Spleens of SCID mice were harvested and splenocytes were seeded in 96-well culture plates at a density of 2×10^5^ cells per well in DMEM containing 10% FBS in the presence or absence of purified human PG (25 µg/ml) as Ag in triplicate wells. Cells were cultured for 5 days, and proliferation was measured on the basis of [^3^H]thymidine incorporation. Results were expressed as stimulation index (SI), which is a ratio of isotope incorporation (cpm) by PG-stimulated and non-stimulated cultures.

Concentrations of PG-specific Abs in the sera of SCID mice were determined by ELISA as described elsewhere [27], [30], [32]. Briefly, MaxiSorp ELISA plates (Nunc, Denmark) were coated with human PG (0.75 µg/well) overnight. Unbound material was washed out, and the wells were blocked with 1.5% fat-free milk in PBS. Serially diluted (1∶100–1∶200,000) serum samples from individual mice, and internal standard samples (pooled serum from arthritic BALB/c mice, containing known amounts of PG-specific IgG1 and IgG2a) were incubated with the immobilized PG. PG-specific IgG1 and IgG2a were detected using HRP-conjugated secondary Abs (Invitrogen), followed by HRP substrate and *o*-phenylene-diamine (Sigma) as chromogen. Optical densities were measured at 490 nm in an ELISA reader. Data were expressed in mg/ml serum (PG-specific IgG1) or µg/ml serum (PG-specific IgG2a).

### Statistical analysis

Results are expressed as the means ± SEM unless noted otherwise. Statistical analysis was performed using GraphPad Prism 6 program (GraphPad Software, La Jolla, CA). For comparison of two groups of data, the parametric Student’s t test or the non-parametric Mann-Whitney U test was employed. Multiple comparisons were performed using the Kruskal-Wallis test followed by Dunn’s multiple comparisons test. Data resulting from repeat measurements over time were analyzed using two-way repeated measures analysis of variance. P values of less than 0.05 were accepted as statistically significant.

## Results

### Murine BM cells cultured in the presence of G-CSF, GM-CSF, and IL-6 give rise to a cell population resembling SF-MDSCs

**Figure 1:**
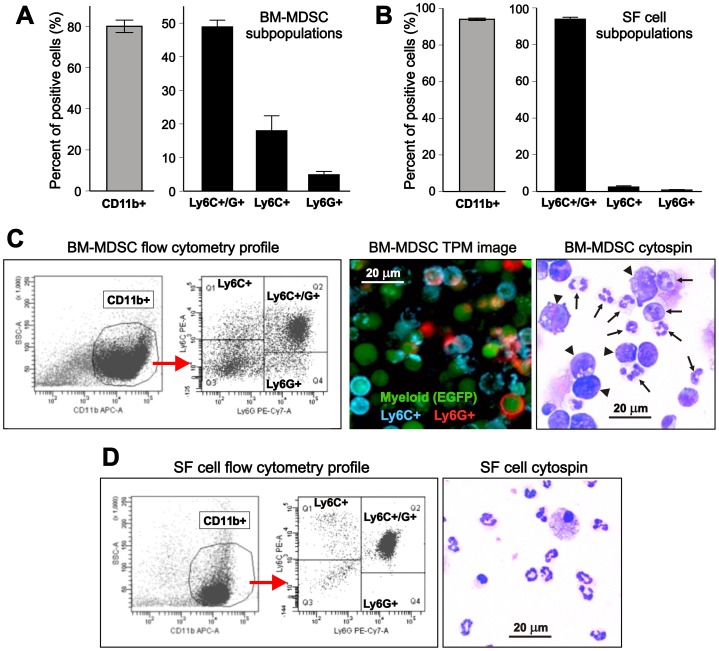
Phenotype and morphology of myeloid-derived suppressor cell (MDSC)-like cells generated in vitro from murine bone marrow (BM) in comparison with synovial fluid (SF) cells. (**A**) Phenotype of MDSCs arising from growth factor-cytokine treated BM cell cultures as determined by flow cytometry. BM cells were cultured in the presence of GM-CSF, IL-6, and G-CSF (10 ng/ml each). On day 3, cells were immunostained for CD11b, Ly6C, and Ly6G. Approximately 80% of the cells expressed the common myeloid marker CD11b (gray bar). Gating on CD11b^+^ cells revealed that the majority of them co-expressed Ly6C (marker of the “monocytic” subset) and Ly6G (marker of the “granulocytic” subset), but cells expressing only one marker were also present (black bars). The results are the means ± SEM of 7 independent BM cultures. (**B**) In SF, the vast majority of the CD11b^+^ myeloid population (gray bar) was found to be cells co-expressing Ly6G and Ly6C, and lower proportions of cells expressed Ly6C or Ly6G only (black bars) than in the BM-MDSC cell cultures. The results are the means ± SEM of 7 separate pools of SF cells freshly harvested from arthritic mouse joints. (**C**) Flow cytometry profile of BM-MDSCs (left panels) is shown as an example of subset identification after gating on CD11b^+^ cells. Fluorescence image of EGFP^+^ BM-MDSCs (middle panel) after surface staining with a blue fluorescent antibody to Ly6C and a red fluorescent antibody to Ly6G shows cells expressing one or both markers. BM for culture was obtained from an EGFP-LysM-Tg mouse expressing EGFP (green fluorescence) in myeloid cells. Imaging was performed using two-photon microscopy (TPM). Morphology of BM-MDSCs (right panel) was visualized by Wright-Giemsa staining of a cytospin preparation, which shows both polymorphonuclear granulocyte (neutrophil)-like cells (arrows) and large precursor-like cells (arrowheads). (**D**) Flow cytometry profile (left) and morphology (right) of SF cells harvested from the arthritic joints of mice with PGIA. While the CD11b^+^ myeloid population is large in both the BM-MDSC culture and arthritic SF, and is dominated by Ly6C/Ly6G double positive cells in both samples (analyzed simultaneously), BM-MDSCs show greater heterogeneity in morphology than SF cells.

The primary goal of this study was to establish a culture method by which BM cells can be enriched in myeloid cells resembling SF-MDSCs in both their phenotype and function. We chose BM because it is the body’s largest reservoir of myeloid precursors from which large numbers of MDSCs can be generated under appropriate conditions. GM-CSF is essential for the survival and suppressor activity of MDSCs [34], and one study reported successful generation of MDSCs from human blood in 7 days with a combination of GM-CSF and IL-6 [35], factors that are also present in the SF of RA patients [36]. In preliminary experiments, we sought to determine whether BM cells cultured for 3 to 7 days in the presence GM-CSF and IL-6 acquire an SF-MDSC-like phenotype. Although the BM culture became enriched in CD11b^+^ cells under this condition as determined by flow cytometry, unlike SF-MDSCs, only a small proportion of these myeloid cells expressed Ly6G, a marker of granulocytic MDSCs (data not shown). We added G-CSF as a booster of the granulocytic lineage to the BM culture, which resulted in the rise of cell populations expressing Ly6G alone, or co-expressing Ly6G (at high levels) with low-to-intermediate levels of the monocytic MDSC marker Ly6C (Fig. 1A, and Fig. 1C [left panel]). This overall phenotype was achieved in 3 days of culture in the presence of GM-CSF, IL-6, and G-CSF (10 ng/ml each); longer culture or higher doses of G-CSF did not result in increases in Ly6G^+^ or double Ly6C^+^Ly6G^+^ cells (data not shown). In comparison with CD11b^+^ SF cells (Fig. 1B and D), BM-MDSCs contained fewer double Ly6C^+^Ly6G^+^ cells and higher proportions of subsets expressing only one of these markers (Fig. 1A and C). However, cells co-expressing Ly6C and Ly6G clearly represented a dominant population in both the BM-MDSC cultures and freshly harvested SF (Fig. 1A–D). Our choice of the combination of growth factors to generate SF-MDSC-like cells from BM was supported by the finding that SF from mice with PGIA contained high amounts of GM-CSF and G-CSF, and detectable amounts of IL-6. In each case, the SF concentrations of these factors exceeded the serum levels (Table S1).

Immunofluorescence staining of BM-MDSC-like cells generated from EGFP-LysM mice (cultured for 3 days as described above), followed by imaging with TPM demonstrated that the majority of myeloid (EGFP^+^) cells expressed either Ly6G or Ly6C, or both markers (Fig. 1C, middle panel). Both polymorphonuclear granulocyte (neutrophil)-like cells (Fig. 1C, right panel: arrows) and large precursor-like cells (Fig. 1C, right panel: arrowheads) were seen in the cytospin preparations of such cells.

Overall, the flow cytometry profile and morphology of BM-MDSC-like cells (Fig. 1C) demonstrated greater heterogeneity than those of fresh SF cells (Fig. 1D), suggesting that in addition to the dominant population of double-positive Ly6G^hi^Ly6C^int/lo^ cells (also present in SF), BM-MDSC cultures contained a variety of immature myeloid cells with intermediate phenotypes.

Ly6G is highly expressed by both mature neutrophils and granulocytic MDSCs in mice [21], [37], and no additional surface markers are available to distinguish between these two types of cells. On the other hand, among monocytic cells, classical (or “inflammatory”) monocytes are characterized by high expression of Ly6C, whereas non-classical (also termed “patrolling” or “anti-inflammatory” monocytes/macrophages) express Ly6C at low levels [15]. We used additional mAbs against monocyte/macrophage markers, including F4/80, CD115, and CD80 to identify distinct subsets within the two monocytic cell categories. However, we found that only a few percent of cultured BM-MDSCs or freshly harvested SF cells expressed F4/80 and CD80, and less than 1% of them was CD115^+^ (Fig. S1). The highest proportions of F4/80^+^ and CD80^+^ cells were detected within the Ly6C^lo/−^ population among BM-MDSCs (4–5%) (Fig. S1A) and in the Ly6C^hi/int^ population among SF cells (0.5–2%) (Fig. S1B). CD115^+^ cells represented 0.2% of both Ly6C^hi/int^ and Ly6C^lo/−^ BM-MDSCs (Fig. S1A), and 0.7% of Ly6C^lo/−^ SF cells (Fig. S1B).

As CD11b^lo/−^Ly6C^hi^CD115^+^ osteoclast precursors have been identified in the BM of mice with inflammatory arthritis [38], we also screened the CD11b^lo/−^ populations of cultured BM-MDSCs and freshly harvested SF for the presence of such osteoclast precursor-like cells. However, we could not detect CD115^+^ cells in either the Ly6C^hi/int^ or the Ly6C^lo/−^ fraction of CD11b^lo/−^ BM-MDSCs (Fig. S2A) or SF cells (Fig. S2B).

### BM-MDSCs have the ability to suppress both Ag/DC-dependent and -independent proliferation of T cells in vitro

**Figure 2:**
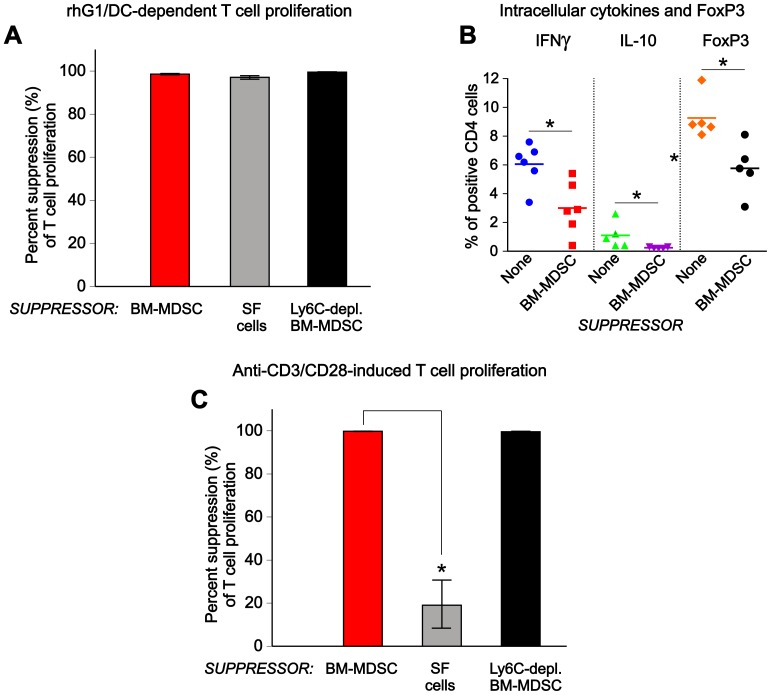
Suppression of antigen (Ag)-specific and non-specific T-cell responses by BM-MDSCs. (**A**) T cells, purified from the spleens of mice expressing a PG-specific T cell receptor transgene (PG-TCR-Tg) were cultured for 5 days with dendritic cells (DCs) loaded with recombinant G1 domain of human PG (rhG1) in the absence or presence of the following “suppressors”: BM-MDSCs (red bar), arthritic SF cells (gray bar), or Ly6C^hi^ (monocytic) cell-depleted BM-MDSCs (black bar). The ability of suppressors to inhibit Ag (rhG1)-specific T-cell proliferation (which is also dependent on Ag presentation by DCs) was assessed on the basis of inhibition of [^3^H]thymidine incorporation by the T cells. Percent suppression was calculated as described in the Methods. All suppressors exhibited robust inhibition of T-cell proliferation. The results shown are from 5 independent experiments. (**B**) T cells from PG-TCR-Tg mice were cultured for 2 days with rhG1-loaded DCs and BM-MDSCs as described for panel A. The percent of CD4^+^ T cells containing IFNγ, IL-10, or FoxP3 (CD4^+^CD25^+^FoxP3^+^ T regulatory cells, Tregs) was determined by flow cytometry. The results shown are the individual values (n = 5–6) and the means. On average, the percentages of IFNγ^+^ cells, IL-10^+^ cells, and Tregs were lower in the presence of BM-MDSCs (*p<0.001, 0.001, and 0.05, respectively; Mann-Whitney U test) than in their absence (None). (**C**) T cells from PG-TCR-Tg mice were cultured in anti-CD3/CD28-coated plates for 4 days in the absence or presence of the listed suppressors. Percent suppression was calculated and results expressed as described for panel A. Non-depleted BM-MDSCs and BM-MDSCs depleted in Ly6C^hi^ cells were equally potent in suppressing anti-CD3/CD28-induced T-cell proliferation, while arthritic SF cells exhibited much weaker inhibition (*p<0.01, n = 5; Kruskal-Wallis test followed by Dunn’s multiple comparisons test) in this induction system.

To study the effect of BM-MDSC-like cells on Ag-specific T-cell proliferation, we cultured Ag (rhG1)-loaded DCs with T cells isolated from the spleens of naive PG-TCR-Tg mice in the presence or absence of BM-MDSCs as suppressors. Additional “suppressors” (as comparators) were SF cells, and BM-MDSCs depleted in Ly6C^hi^ cells. Ag-dependent T-cell proliferation was dramatically reduced in the presence of BM-MDSCs, i.e., BM-MDSC-mediated suppression reached nearly 100% (Fig. 2A, red bar). Compared with SF cells (Fig. 2A, gray bar) BM-MDSCs were equally potent in suppressing T-cell proliferation. As also reported for SF cells [21], depletion of the Ly6C^hi^ monocytic subset from the BM-MDSCs (Fig. 2A, black bar) did not reduce their suppressive capacity. BM-MDSC-mediated suppression of Ag-specific T-cell proliferation was accompanied by significant decreases in the percentage of CD4^+^ T helper (Th) cells containing intracellular cytokines (IFNγ in Th1 and IL-10 in Th2 cells) as well as in the percentage of Tregs (CD4^+^CD25^+^ cells containing FoxP3) (Fig. 2B).

Since we found previously that SF-MDSCs from arthritic mice suppressed the maturation and Ag presenting capacity of DCs [21], we investigated the effect of BM-MDSCs on the expression levels of DC maturation markers MHC II and CD86. However, we could not detect significant changes in the expression level of either marker in DCs upon co-culture with BM-MDSCs (Fig. S3). Since BM-MDSCs failed to decrease the surface expression of these molecules by the DCs, this experiment also suggested that the observed suppression of the proliferation and cytokine/FoxP3 content of T cells was not due to release of cytotoxic substances from the BM-MDSCs.

To determine whether the suppressive effect of BM-MDSCs on T-cell proliferation was Ag-dependent (for which the presence of DCs was required) or Ag-independent, we stimulated the PG-TCR-Tg T cells with anti-CD3 and anti-CD28 mAbs in the presence or absence of BM-MDSCs. In this Ag/DC-independent system, BM-MDSCs also exhibited potent suppressor activity in (Fig. 2C, red bar), whereas suppression by SF cells was very weak (Fig. 2C, gray bar), consistent with our previous report [21]. As expected, depletion of Ly6C^hi^ cells did not reduce the capacity of BM-MDSCs to suppress the anti-CD3/CD28-induced proliferation of T cells (Fig. 2C, black bar).

### The suppressive effects of BM-MDSCs on T cells can be reversed by iNOS inhibitors in vitro

**Figure 3:**
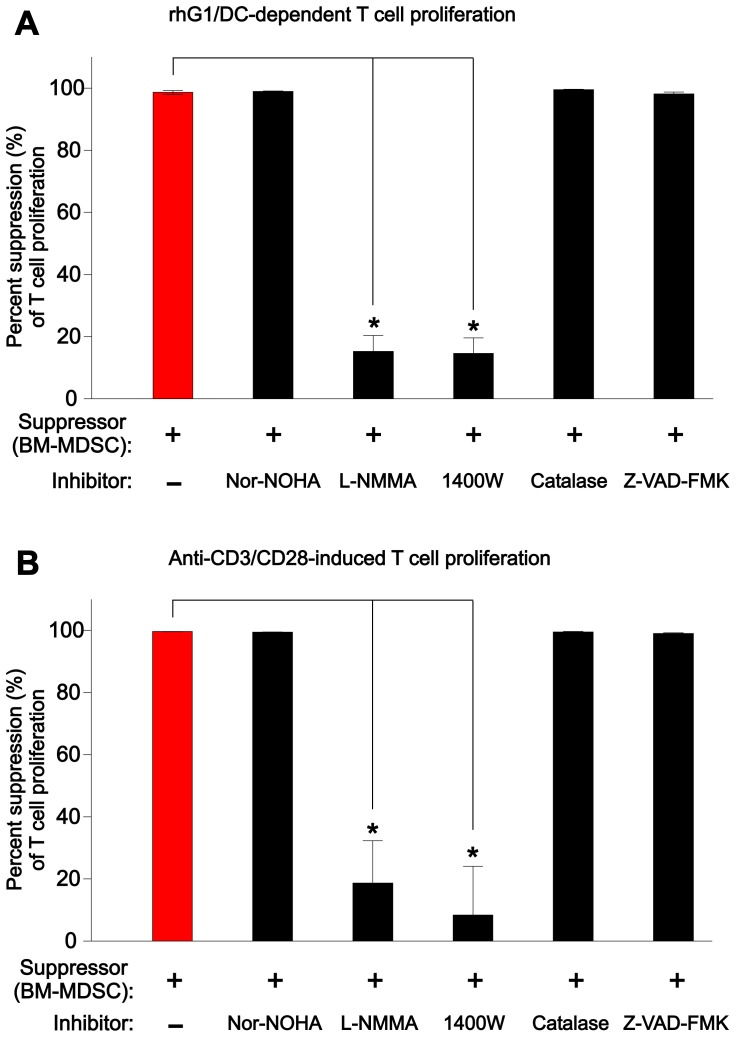
Reversal of the suppressive effect of BM-MDSCs on T-cell proliferation by inhibitors of inducible nitric oxide synthase (iNOS). Various inhibitors of MDSC effector molecules, including the arginase 1 inhibitor nor-NOHA, iNOS inhibitors L-NMMA and 1400W, the reactive oxygen species (ROS) scavenger catalase, and the caspase/apoptosis inhibitor Z-VAD-FMK, were used to inhibit the BM-MDSC-mediated suppression of (**A**) Ag (rhG1)-induced/DC-dependent and (**B**) anti-CD3/CD28-induced proliferation of PG-TCR-Tg T cells. The results (compiled from 2 independent series of experiments, each with 2 co-cultures) are expressed as percent suppression of T-cell proliferation in the presence (black bars) or absence (red bar) of inhibitors. While suppression of T-cell proliferation in both induction systems was significantly reversed by the iNOS inhibitors L-NMMA and 1400W (*p<0.0001 in all cases; Kruskal-Wallis test followed by Dunn’s multiple comparisons test), none of the other inhibitors had a significant effect on BM-MDSC-mediated suppression of T cells.

To reveal the possible mechanism of the suppressive activity of the BM-MDSCs, we repeated the Ag-dependent and Ag-independent T-cell proliferation assays with and without various inhibitors of MDSC-related effector molecules such as arginase 1 (nor-NOHA), iNOS (L-NMMA and the more selective 1400W), and ROS (catalase). A caspase (apoptosis) inhibitor (Z-VAD-FMK) was used as a MDSC-unrelated control. Both Ag (rhG1)- and anti-CD3/CD28-induced T-cell proliferation remained suppressed in the presence of the arginase 1 inhibitor, the ROS scavenger, or the caspase inhibitor (Fig. 3A and B). However, BM-MDSCs lost much of their ability to suppress T-cell proliferation in both induction systems in the presence of the iNOS inhibitors (Fig. 3), suggesting that the main MDSC product mediating T-cell suppression was NO.

### BM-MDSCs exhibit upregulated iNOS expression and elevated NO production

**Figure 4:**
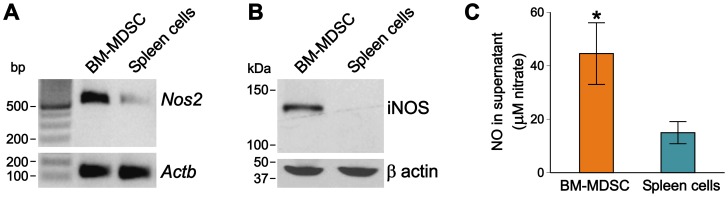
Expression and activity of iNOS in BM-MDSCs. (**A**) Comparison of murine iNOS (*Nos2*) transcript levels in BM-MDSCs and spleen cells revealed that iNOS mRNA was upregulated in BM-MDSCs. The housekeeping gene (*Actb*, encoding β-actin) was expressed at equal levels. Results of one of 2 replicate experiments (with similar results) are shown. (**B**) Western blot using an antibody against murine iNOS demonstrated the presence of iNOS protein in BM-MDSCs, but not in spleen cells. The β-actin control blot shows equal sample loading. One of 3 independent Western blots is shown. (**C**) iNOS activity was assayed on the basis of NO release into the supernatants of cultures containing BM-MDSCs (orange bar) or spleen cells (green bar), and expressed as total nitrate concentration (µM). BM-MDSC-containing cultures produced significantly higher amounts of NO than spleen cells did (*p<0.05, n = 5 cultures/cell type; Mann-Whitney U test). Molecular markers: bp, base pairs; kDa, kilodalton.

To corroborate the results of T-cell proliferation assays indicating a role for NO in the suppressor activity of BM-MDSCs, we performed RT-PCR to assess expression of iNOS (*Nos2*) mRNA in BM-MDSCs in comparison with spleen cells harvested from arthritic mice [21]. BM-MDSCs demonstrated significant up-regulation of *Nos2* mRNA as compared with spleen cells (Fig. 4A), while the housekeeping gene (*Actb*, encoding β-actin) was expressed at equal levels. The results of Western blot were consistent with the results of RT-PCR, showing a large amount of iNOS protein (∼130 kDa) in BM-MDSCs, but not in spleen cells (Fig. 4B).

The enzymatic activity of iNOS was assessed by measuring nitrite/nitrate concentrations (as indicators of NO production) in supernatants of BM-MDSCs (cultured in the presence of DCs and rhG1 with or without T cells) and spleen cell cultures. Consistent with the iNOS expression data, much higher levels of NO were detected in the supernatants of BM-MDSCs-containing cultures (Fig. 4C, orange bar) than in those of spleen cell cultures (Fig. 4C, green bar).

### Injection of BM-MDSCs into SCID mice reduces Ag-specific immune responses and ameliorates adoptively transferred arthritis

**Figure 5:**
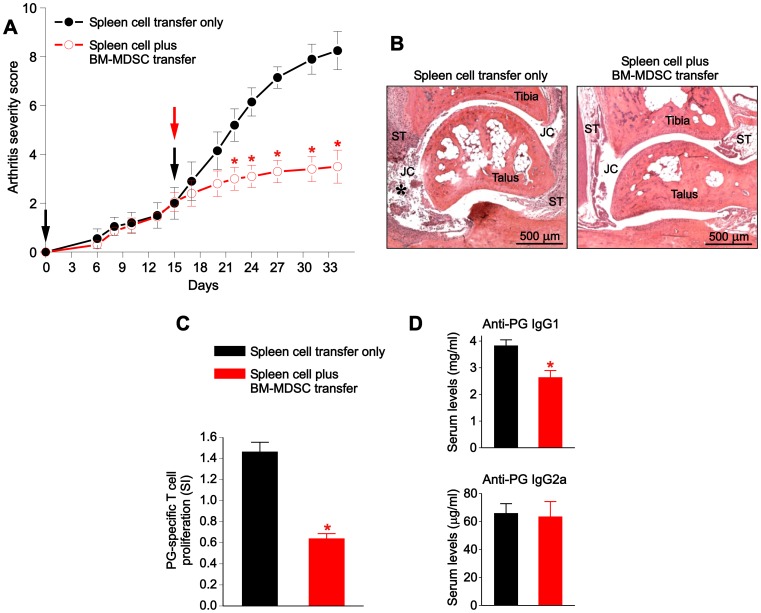
Effects of BM-MDSCs on arthritis severity and Ag (PG)-specific immune responses in SCID mice with PGIA. (**A**) Effect of BM-MDSC transfer on arthritis severity. Arthritis was induced in SCID mice via 2 transfers of spleen cells (black arrows) from wild type mice with PGIA as described in the Methods. At the early phase of arthritis, one group of the SCID recipients was co-injected with BM-MDSCs (red arrow). Disease severity scores were monitored until day 34. Arthritis progressed rapidly in the control group (black line), but not in the BM-MDSC-treated group (red line) (*p<0.05, n = 10 mice/group; two-way repeated measures analysis of variance). (**B**) Joint histopathology of control (left panel) and BM-MDSC-treated (right panel) mice on day 34. The ankle joint of the control mouse demonstrated massive leukocyte infiltration (star) in the joint cavity (JC) and synovial tissue (ST) as well as synovial hyperplasia. The articulating surfaces appeared rough due to cartilage damage. In the ankle joint of the BM-MDSC-treated mouse only mild synovial hyperplasia was seen, suggesting the resolution of initial (previous) inflammation. Representative hematoxylin-eosin-stained tissue sections from both groups are shown. (**C**) Antigen (PG)-specific T-cell responses of control and BM-MDSC-treated mice. T-cell responses were compared between the two groups on day 34 by measuring spleen cell proliferation in the presence or absence of PG in vitro. Results are expressed as stimulation index (SI), a ratio of [^3^H]thymidine incorporation by PG-stimulated and non-stimulated cultures. The SI of the BM-MDSC-injected group (red bar) was significantly lower than the SI of the control group (black bar) (*p<0.0001, n = 10 mice/group; Student’s t test). (**D**) Serum levels of anti-PG antibodies in the control and BM-MDSC-treated groups as determined by ELISA. The levels of IgG1 anti-PG antibodies (top) were significantly lower in the sera of BM-MDSC-injected mice than in control mice (*p<0.01, n = 5 samples/group; Mann-Whitney U test), while the levels of IgG2a anti-PG antibodies (bottom) were similar.

To test whether BM-MDSCs could affect the development of arthritis, an adoptive transfer model of PGIA was employed. On day 0, spleen cells from arthritic wt BALB/c donor mice were injected with Ag (human PG) into SCID recipients. When the clinical signs of arthritis started to develop (15 days after the first injection), the SCID mice were divided into 2 groups with similar mean disease scores, and a second injection was administered. The first (control) group received only arthritic spleen cells and PG, while the second group received the same plus BM-MDSCs. Arthritis severity scores in the control group increased further (Fig. 5A, black line), while, in sharp contrast, the scores of SCID mice transferred with BM-MDSCs remained low until the end (day 34) of the monitoring period (Fig. 5A, red line). Histopathology revealed massive leukocyte infiltration and synovial hyperplasia as well as cartilage erosion in the ankle (tibio-talar) joints of control SCID mice transferred with spleen cells from arthritic donors (Fig. 5B, left panel). In contrast, only mild synovial hyperplasia was observed without evidence of gross inflammation or cartilage damage in the ankle joints of SCID mice co-transferred with spleen cells and BM-MDSCs (Fig. 5B, right panel).

To determine whether the BM-MDSC-mediated protection from arthritis progression was associated with reduced Ag-specific T-cell responses and Ab production, we compared the PG-specific T-cell responses and serum IgG1and IgG2a Ab levels in control and BM-MDSC-injected SCID mice. PG-specific T-cell proliferation was significantly lower in the BM-MDSC-injected group (Fig. 5C). Serum levels of IgG1 isotype (but not of IgG2a isotype) anti-PG Abs were also significantly reduced in the BM-MDSC recipient group (Fig. 5D).

**Figure 6:**
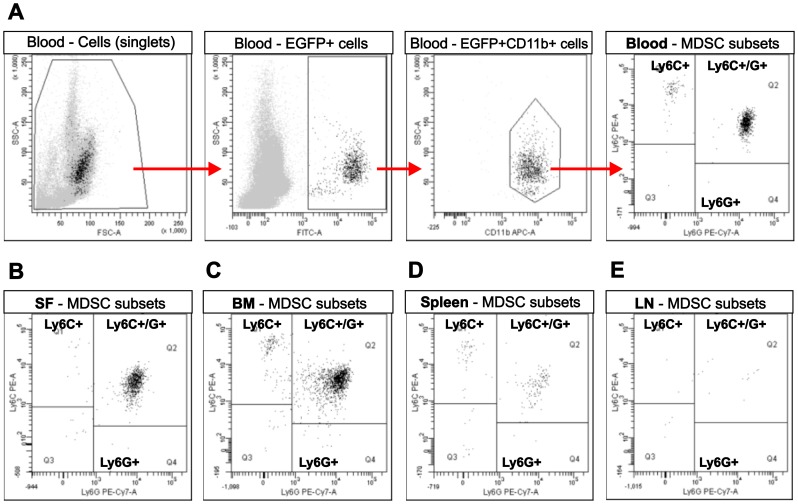
Tissue distribution of EGFP^+^ BM-MDSCs injected into SCID mice with adoptively transferred PGIA. BM-MDSCs, generated from EGFP-LysM-Tg mice (expressing EGFP in myeloid cells only) were co-injected with arthritic spleen cells into SCID mice at the early phase of adoptively transferred PGIA, as described in the Methods. To assess the tissue distribution of fluorescent donor cells, 19 days after the co-transfer of the cells (**A**) peripheral blood, (**B**) synovial fluid (SF), (**C**) BM, (**D**) spleen, and (**E**) joint-draining lymph nodes (LN) were harvested from the SCID recipients, immunostained, and subjected to flow cytometry. The gating strategy, as demonstrated on the blood cells (top panels), involved gating first on single cells, then on EGFP^+^ cells, followed by gating on the CD11b^+^ myeloid population (red arrows). Subset composition of EGFP^+^CD11b^+^ cells was determined on the basis of Ly6C and Ly6G expression. Peripheral blood, SF, and BM contained very well detectable populations of Ly6C^int^Ly6G^hi^ (granulocytic) cells and much smaller populations of Ly6C^hi^Ly6G^−^ (monocytic) cells. Cells belonging to either subset were less frequent in the spleen, and nearly undetectable in the LNs. Representative flow cytometry dot plots of cells from 1 of 3 mice (except for SF, which was pooled from all of the 3 mice) are shown.

In a separate experiment, we assessed the distribution and subset composition of transferred EGFP^+^ BM-MDSCs in various fluids and tissues (blood, SF, BM, spleens, and LNs) of SCID mice with adoptively transferred PGIA (induced as described above) 19 days after BM-MDSC injection. The donor EGFP^+^ BM-MDSCs (injected at half of the optimal therapeutic dose) were found in considerable amounts in the blood (Fig. 6A), SF (Fig. 6B), and BM (Fig. 6C) of the recipient mice. The spleen (Fig. 6D) contained a much lower percentage of these cells, and the LNs (Fig. 6E) were virtually free of BM-MDSCs. In each tissue or fluid, the granulocytic subset (Ly6G^hi^Ly6C^int^) dominated, although small populations of monocytoid (Ly6C^hi^Ly6G^−^) MDSCs were also present (Fig. 6).

## Discussion

MDSCs have been described as innate immune cells with a remarkable capacity to control adaptive immune responses [8]. Although first described in tumor-bearing animals and cancer patients [5], [39], MDSCs have been recently identified in a variety of autoimmune conditions [14], [18]–[20] that are characterized by excessive activation of the adaptive immune system.

We have reported previously that the SF of mice with PGIA, an animal model of RA, contains a population of cells that meets the criteria of MDSCs [21]. Our data suggested that the inflamed joint in PGIA is a supportive microenvironment in which myeloid cells survive and acquire a mainly granulocytic MDSC-like phenotype and potent suppressor activity toward DCs and Ag-specific T cells. By limiting the expansion of pathogenic T cells and the maturation of DCs locally or in lymphoid organs, MDSCs present in the SF could suppress local inflammation or prevent the spreading of arthritis to other joints. This hypothesis would be best tested by transferring SF-MDSCs to mice at the early phase of PGIA. However, the number of cells that can be collected from murine SF is limited, and SF-MDSCs do not expand in culture [21]. Thus, we sought an alternative source of cells to generate large quantities of MDSCs for potential therapeutic intervention (via cell transfer) in PGIA. Studies by others reported accumulation of MDSCs in the spleens of mice with cancer [13] or autoimmune diseases [14], [20]. However, previously we found that in mice with PGIA, the splenic myeloid population was modest in size and lacked suppressor activity [21]. Because the BM contains considerable amounts of myeloid precursors, it appeared to be a plausible source of cells with a potential to become SF-MDSC-like cells under appropriate culture conditions.

Enrichment of murine BM in immature myeloid cells in vitro in the presence of GM-CSF was described before, but these cells showed a tendency to become myeloid DCs if no other factor was added [40]. On the other hand, Lechner et al. [35] reported generation of cells with monocytic MDSC phenotype and immunosuppressive capacity by culturing normal human PBMCs in the presence of GM-CSF and IL-6. As we have shown in this study, murine BM cells treated with a combination of GM-CSF, IL-6, and G-CSF for 3 days give rise to a dominant population of Ly6G^hi^Ly6C^int/lo^ granulocytic SF-MDSC-like cells, although such cultures also contain smaller populations of cells with intermediate phenotypes. Further phenotypic analysis of the monocytic subset using mAbs to monocyte/macrophage markers F4/80, CD115, and CD80 failed to detect a distinct population expressing these markers, although approximately 5% of BM-MDSCs in the Ly6C^lo/−^ fraction could be defined as CD11b^+^F4/80^+^CD80^+^ macrophages. We found a small population (0.7%) of cells expressing CD115, the receptor for macrophage colony stimulating factor among CD11b^+^Ly6C^lo/−^ SF cells. A previous study reported increased number of Ly6C^hi^CD115^+^ osteoclast precursor cells in the BM of mice with inflammatory arthritis [38]. Such precursors were identified within the CD11b^lo/−^ population of BM cells and also had myeloid suppressor activity [38]. In our case, CD11b^lo/−^Ly6C^hi/int^ fractions of both BM-MDSCs and SF were devoid of CD115^+^ cells. Although we cannot rule out the possibility that the few CD115^+^ cells found in the CD11b^+^Ly6C^lo/−^ SF population may differentiate into mature osteoclasts, the findings suggest that neither the BM-MDSC culture condition nor the SF milieu is conducive to the development of CD11^lo/−^Ly6C^hi^CD115^+^ osteoclast precursors.

We have suggested previously [21] that MDSC precursors entering the joints may acquire a maturation-resistant phenotype in a milieu rich in myelopoietic growth factors and cytokines. Indeed, we found high levels of GM-CSF and G-CSF, and detectable levels of IL-6 in the cell-free SF of mice with PGIA. Similar to the findings reported by Wright et al. [36] in SF and serum samples from RA patients, we detected much higher levels of these factors in the SF than in the serum of mice with PGIA. These observations indicate that GM-CSF, G-CSF, and IL-6 (besides other pro-inflammatory mediators) are produced locally by joint-resident cells in both RA and PGIA, and likely support the survival and suppressor activity of MDSCs in the SF.

The MDSC-like cells that we generated from murine BM under the conditions described were true MDSCs, as they exerted profound suppressive effects on both the Ag-specific and non-specific (polyclonal) proliferation of T cells in vitro. While both BM-MDSCs and SF cells inhibited the expansion of Ag-stimulated T cells to a comparable degree, BM-MDSCs were much more potent than SF cells in suppressing anti-CD3/CD28-induced polyclonal T-cell proliferation. This suggests that the suppressive ability of SF cells is selective, while BM-MDSCs are capable of inhibiting T-cell responses to either Ag-specific or non-specific stimuli. With regard to BM-MDSCs, our finding is consistent with a recently published study [41] in which CD11b^+^Gr-1^+^ “immature” myeloid cells, isolated from the BM of normal mice, have been found to suppress the Ag-independent (anti-CD3/CD28- or mitogen-induced) proliferation of T cells.

Although it has been suggested that Ly6G^−^Ly6C^hi^ monocytic MDSCs suppress T-cell activity more strongly than the granulocytic subset [34], we found that upon depletion of Ly6C^hi^ subpopulation, the BM-MDSCs retained their suppressive ability toward T cells. This supports our previous [21] and other authors’ [14], [20] conclusions that Ly6G^+^ granulocytic MDSCs represent a subset with potent suppressor activity.

Our experiments elucidating the molecular mechanisms of BM-MDSC-mediated suppression revealed that inhibitors of iNOS were able to reverse both the Ag-specific and non-specific suppression of T-cell proliferation. Consistent with this observation, iNOS was upregulated in BM-MDSCs at both mRNA and at protein levels, and NO was present in high quantities in the supernatants of BM-MDCS cultures. NO can suppress T-cell function via multiple mechanisms including chemical alteration of the TCR and inhibition of kinases and transcription factors involved in the IL-2 receptor signaling pathway [8], [42]. Granulocytic MDSCs have been shown to exert suppression on T cells via an arginase 1-dependent [20] or ROS-dependent [13] mechanism. However, MDSCs with a granulocytic phenotype that are present in the SF of arthritic mouse joints [21] or in the BM [41] as well as those generated from murine BM ex vivo (this study) are clearly capable of inhibiting T-cell responses in a NO-dependent manner. In relevance to RA, elevated concentrations of NO were found in the serum and SF of RA patients with the SF levels exceeding those in serum, suggesting NO production locally in the joint [43]. Since cells with a granulocytic phenotype constitute the major cell population in RA SF [44], [45], they could be the primary source of NO, thus functioning as local granulocytic MDSCs.

Although detailed characterization of the T-cell signaling pathways altered by BM-MDSCs was beyond the scope of our investigations, intracellular levels of IFNγ and IL-10 in CD4^+^ Th cells and the induction of Tregs were assessed. Intracellular concentration of IFNγ, the pro-inflammatory cytokine produced by the Th1 subset of CD4^+^ cells, was reduced by BM-MDSCs, but so was the anti-inflammatory Th2 cell-derived cytokine IL-10. Although MDSC-mediated induction of Treg cell differentiation has been reported in vitro and in tumor-bearing mice [46], we found that the proportion of Treg cells was actually reduced in the presence of BM-MDSCs. Our observations suggest that the suppressive effect of BM-MDSCs is not selective and may extend to several T cell subsets.

Studies have reported successful intervention in various diseases by in vivo transfer of MDSCs. Highfill et al. [47] generated MDSCs in vitro from the BM of tumor-free mice in the presence of GM-CSF, G-CSF, and IL-13. Such cells inhibited responses to allogeneic cells in vitro and in graft-versus-host disease [47]. In an animal model of inflammatory bowel disease, transfer of sorted CD11b^+^Gr-1^+^ cells abrogated enterocolitis, indicating a direct immune regulatory effect via NO production [17]. In another study, it was found that MDSCs significantly delayed or prevented type I diabetes onset by suppressing autoreactive T cells and inducing the differentiation of Tregs [19]. In a mouse model of MS, transfer of spleen-derived granulocytic MDSCs delayed the onset and reduced the severity of nervous system disease through suppression of encephalitogenic Th1 and Th17 cells [14]. More recently, Fujii et al. [20] reported accumulation of MDSCs (mainly of the granulocytic phenotype) in the spleens of mice with CIA at the peak of the disease. This finding is congruent with our previous observation that MDSCs accumulate in autoimmune arthritis, although we identified suppressive MDSCs in the SF, not in the spleens, of mice with PGIA [21]. Granulocytic MDSCs, isolated from the spleens of mice with CIA suppressed anti-CD3/CD28-induced T-cell proliferation, but their effects on Ag (type II collagen)-specific immune responses were not investigated [20]. In PGIA, SF-MDSCs exerted suppression on T cells in an Ag-specific manner and were not effective in the Ag-independent system, whereas CD11b^+^ myeloid cells isolated from the spleens at the peak of PGIA were not suppressive in either of these in vitro settings [21]. As described in the present study, MDSCs generated from the BM of naïve mice were able to suppress both Ag-specific and non-specific T-cell responses. These apparent discrepancies may be explained by the functional heterogeneity of MDSCs [10]. It is likely that distinct and overlapping modes of suppressive ability exist, depending not only on the experimental model studied, but also on the specific cytokine milieu supporting and fine-tuning the MDSCs.

Using the adoptively transferred model of PGIA, we found that a single injection of BM-MDSCs into SCID mice after the first signs of arthritis suppressed disease progression and prevented further joint damage. In order to determine whether BM-MDSCs exerted immune modulatory effects in vivo, Ag-specific T-cell proliferation and serum Abs were measured in the recipient mice. The results confirmed that both T- and B-cell responses were significantly inhibited in the BM-MDSC-injected group of mice. In vivo tracking of transferred BM-MDSCs revealed that these MDSCs preferentially accumulated in the BM and SF, sites where their survival was best supported by locally produced myelopoietic growth factors and cytokines. The presence of MDSCs in the blood 19 days after their transfer also indicated active trafficking of these cells between the BM and SF. It is likely, therefore, that BM-MDSCs suppressed arthritis progression by inhibiting the expansion of pathogenic T cells in the BM, the peripheral joints, and, to a lesser degree, in the secondary lymphoid organs of recipient mice.

It was reported earlier that transplantation of syngeneic BM restored immune homeostasis and reduced arthritis severity in mice with PGIA [48]. In this particular case, BM transfer was associated with accumulation of Treg cells in the recipient mice. Although it was not clear if the Treg cells were of donor or recipient origin, the suggested mechanism of disease suppression was a BM-mediated induction of Treg differentiation [48]. While the BM may act as a reservoir of Treg cells [49], it also contains significant amounts of MDSCs and their precursors [41], [49]. It is likely, therefore, that BM-derived MDSCs contributed to the reduction of autoimmune responses and disease severity upon BM transplantation into mice with PGIA.

A recent study reported increased frequency of MDSC-like cells in the peripheral blood of RA patients as compared with the blood of healthy control individuals, but the suppressive properties of these cells were not tested [50]. Most recently, we identified granulocytic MDSCs in the SF of RA patients; these RA SF-MDSCs moderately suppressed the anti-CD3/CD28-induced proliferation of autologous T cells, but potently suppressed alloAg-induced T-cell proliferation in vitro [51]. It is likely that SF-MDSCs inhibit the expansion of joint-homing (pathogenic) T cells in both RA and animal models of the disease. Notably, SF of the arthritic joints of both RA patients and mice has been shown to contain very low proportions of T cells [2], [27], [44], [52], [53]. In addition, T cells isolated from the SF of RA patients exhibit hypo-responsiveness to mitogenic stimuli as compared to blood T cells of the same patients [2], [54]. It is possible, therefore, that SF-MDSCs limit the expansion of T cells locally, thus contributing to the resolution of joint inflammation. Indeed, it was reported that in vivo depletion of MDSCs (using the anti-Gr-1 mAb RB6-8C5) delayed the resolution of arthritis in mice with CIA [20]. However, upon entering the joints at the early phase of arthritis, MDSCs may also cause collateral tissue damage through the release of NO and other noxious products, thereby acting as a “double-edged sword” [9]. Elucidation of the properties and function of MDSCs present in RA patients at distinct anatomical sites (e.g., peripheral blood, BM, SF, and secondary lymphoid organs) would greatly advance our understanding of the role of these cells in the regulation of autoimmunity and joint pathology in RA.

In summary, herein we describe an in vitro method for generating large quantities of MDSCs from murine BM in a controlled and reproducible manner. We show that murine BM-MDSCs, partially resembling MDSCs present in the SF of mice with PGIA, potently inhibit T-cell responses in vitro and in vivo. These results provide insights into an innate control mechanism that is involved in the regulation of immune responses and arthritis severity in an animal model of RA and most likely in human patients as well. Although further studies are warranted, our results also suggest that in vitro enrichment of the BM in MDSCs could improve the therapeutic efficacy of autologous BM transplantation [3] in patients with severe, treatment-resistant RA.

## Supporting Information

Figure S1 **Analysis of monocyte/macrophage marker expression in BM-MDSC-like and SF-MDSC-like cells.** The CD11b^+^ myeloid populations of (**A**) BM-MDSC-like cells and (**B**) SF cells were analyzed by flow cytometry for cells expressing the monocyte/macrophage markers F4/80, CD115, and CD80. (**A**) F4/80^+^ and CD80^+^ cells were more frequent among CD11b^+^Ly6C^lo/−^ than CD11b^+^Ly6C^hi/int^ BM-MDSCs, but very few CD115^+^ cells were detected in either of these populations. (**B**) SF contained much fewer F4/80^+^ and CD80^+^ cells within both the CD11b^+^Ly6C^hi/int^ and CD11b^+^Ly6C^lo/−^ fractions, but slightly more CD115^+^ cells within the CD11b^+^Ly6C^lo/−^ population than BM-MDSCs. Initial gating on CD11b^+^ cells is indicated by red arrows. For subsequent gating, the horizontal line was set to separate the Ly6C^hi/int^ and Ly6C^lo/−^ populations, and the vertical lines were set at the highest levels of background staining with fluorochrome-tagged control IgGs matching the isotypes of F4/80, CD115, and CD80 mAbs. The representative samples show flow dot plots of cells from 1 of 5 independent BM-MDSC cultures, and from 1 of 3 separate pools of SF cells.
(TIF)

Figure S2 **Screening of BM-MDSCs and SF cells for the presence of osteoclast precursor-like cells.** Flow cytometry analysis was performed on the same (**A**) BM-MDSC and (**B**) SF samples described in Figure S1, but with gating on CD11b^lo/−^ cells (red arrows) containing putative Ly6C^hi^CD115^+^ osteoclast precursors. CD115^+^ osteoclast precursor-like cells were not detected in either the Ly6C^hi/int^ or Ly6C^lo/−^ fraction of (**A**) CD11b^lo/−^ BM-MDSCs (**B**) or CD11b^lo/−^ SF cells. The representative samples show flow dot plots of cells from 1 of 5 independent BM-MDSC cultures, and from 1 of 3 separate pools of SF cells.
(TIF)

Figure S3 **Effects of BM-MDSCs of the expression levels of dendritic cell (DC) maturation markers MHC II and CD86.** DCs and BM-MDSCs were generated from BM as described in the Methods. DCs were cultured for 3 days with or without BM-MDSCs. The densities of major histocompatibility complex class II (MHC II) and CD86 maturation markers on the surface of DCs (CD11c^+^ cells) were determined by flow cytometry and the results expressed as mean fluorescence intensity (MFI). (**A**) Expression level of MHC II on the DCs (open bar) slightly increased in the presence of BM-MDSCs (closed bar), but this increase did not reach statistical significance (ns, not significant; p = 0.059; Mann-Whitney U test). (**B**) There was no significant difference in the expression level of CD86 on the DCs either when these cells were cultured without (open bar) and with (closed bar) BM-MDSCs (ns; p = 0.667; Mann-Whitney U test). Data shown are from 5 independent experiments.
(TIF)

Table S1 **Concentrations of GM-CSF, IL-6, and G-CSF in synovial fluid (SF) and serum collected from arthritic (PGIA) mice.**
(DOCX)
