# Supplementary figures and images for: Suppression of Proteoglycan-Induced Autoimmune Arthritis by Myeloid-Derived Suppressor Cells Generated In Vitro from Murine Bone Marrow
Source: PLoS One. 2014 Nov 4;9(11):e111815. doi: 10.1371/journal.pone.0111815 (PMC4219784; doi:10.1371/journal.pone.0111815)

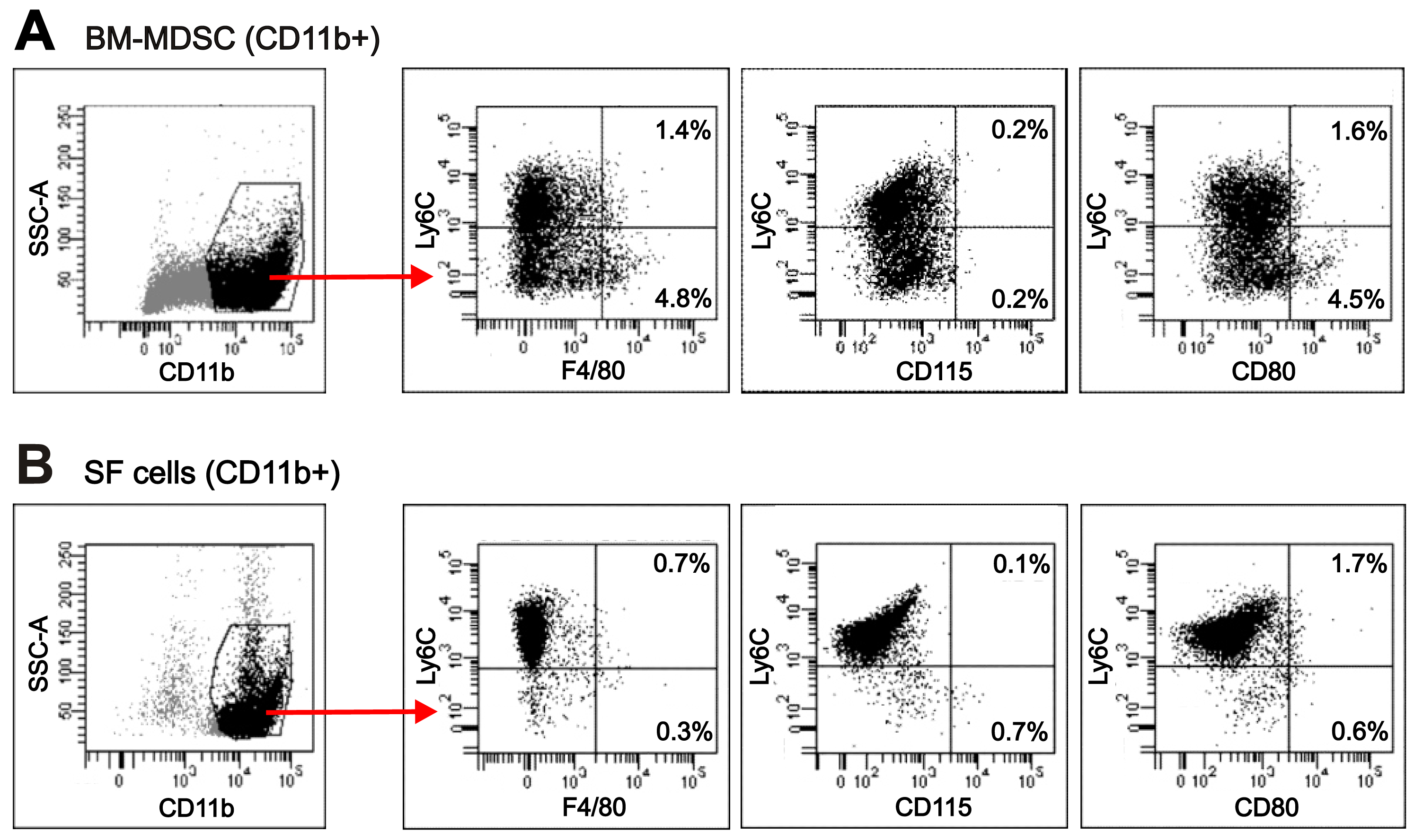

Supplement: Figure S1 — Analysis of monocyte/macrophage marker expression in BM-MDSC-like and SF-MDSC-like cells. The CD11b+ myeloid populations of (A) BM-MDSC-like cells and (B) SF cells were analyzed by flow cytometry for cells expressing the monocyte/macrophage markers F4/80, CD115, and CD80. (A) F4/80+ and CD80+ cells were more frequent among CD11b+Ly6Clo/− than CD11b+Ly6Chi/int BM-MDSCs, but very few CD115+ cells were detected in either of these populations. (B) SF contained much fewer F4/80+ and CD80+ cells within both the CD11b+Ly6Chi/int and CD11b+Ly6Clo/− fractions, but slightly more CD115+ cells within the CD11b+Ly6Clo/− population than BM-MDSCs. Initial gating on CD11b+ cells is indicated by red arrows. For subsequent gating, the horizontal line was set to separate the Ly6Chi/int and Ly6Clo/− populations, and the vertical lines were set at the highest levels of background staining with fluorochrome-tagged control IgGs matching the isotypes of F4/80, CD115, and CD80 mAbs. The representative samples show flow dot plots of cells from 1 of 5 independent BM-MDSC cultures, and from 1 of 3 separate pools of SF cells. (TIF) [file pone.0111815.s001.tif]

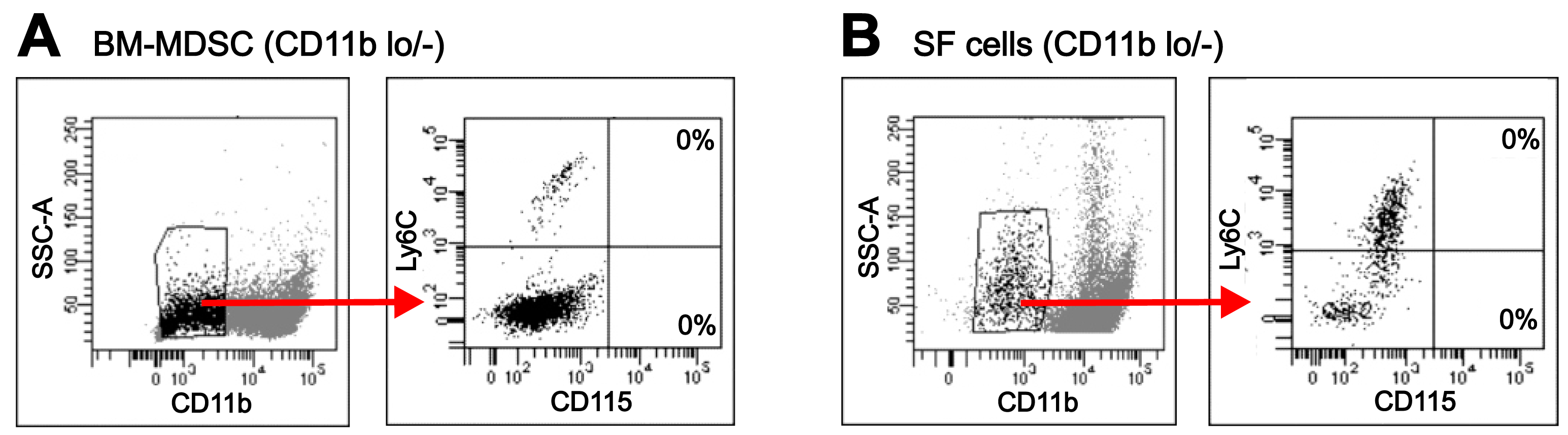

Supplement: Figure S2 — Screening of BM-MDSCs and SF cells for the presence of osteoclast precursor-like cells. Flow cytometry analysis was performed on the same (A) BM-MDSC and (B) SF samples described in Figure S1, but with gating on CD11blo/− cells (red arrows) containing putative Ly6ChiCD115+ osteoclast precursors. CD115+ osteoclast precursor-like cells were not detected in either the Ly6Chi/int or Ly6Clo/− fraction of (A) CD11blo/− BM-MDSCs (B) or CD11blo/− SF cells. The representative samples show flow dot plots of cells from 1 of 5 independent BM-MDSC cultures, and from 1 of 3 separate pools of SF cells. (TIF) [file pone.0111815.s002.tif]

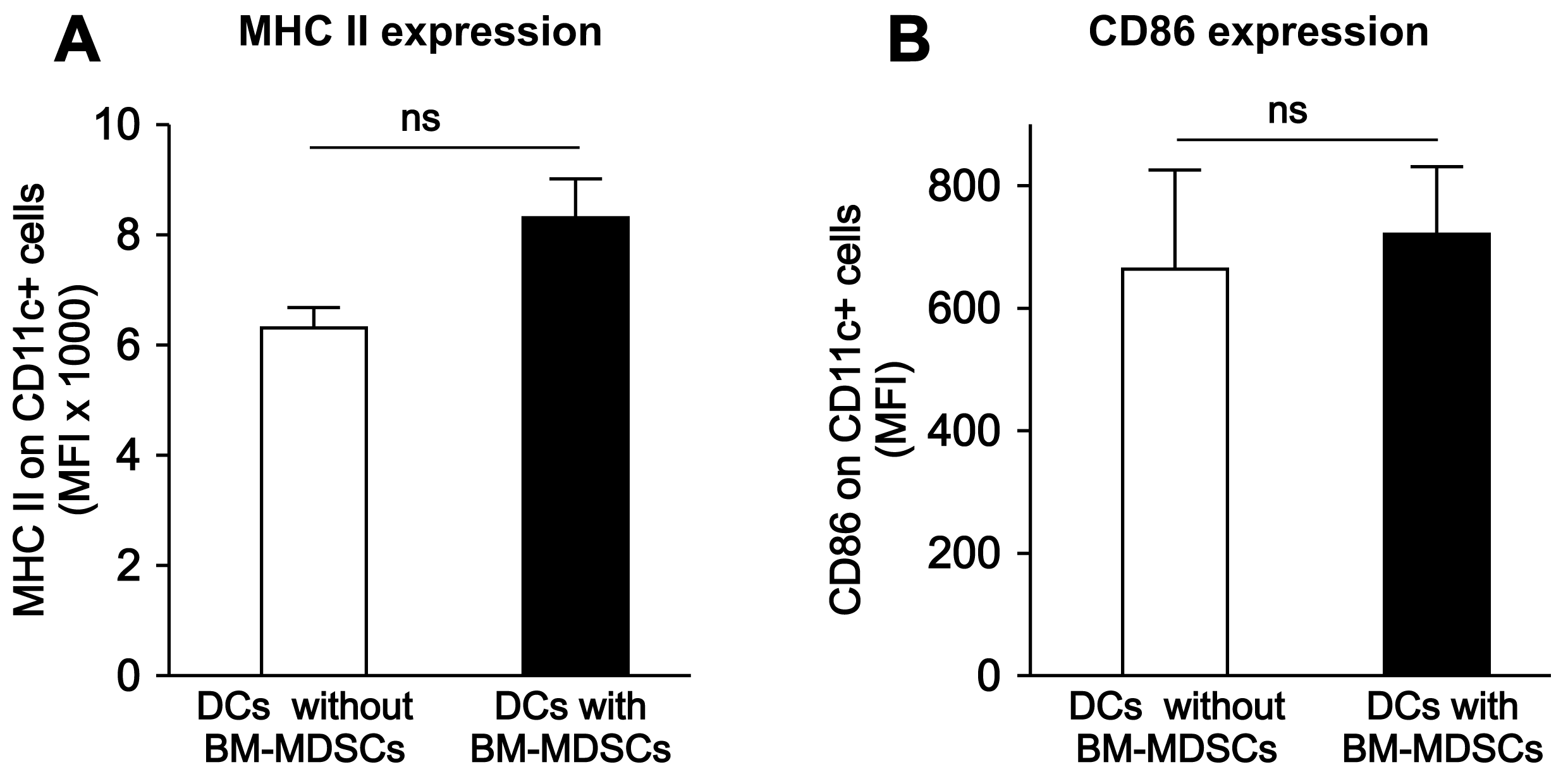

Supplement: Figure S3 — Effects of BM-MDSCs of the expression levels of dendritic cell (DC) maturation markers MHC II and CD86. DCs and BM-MDSCs were generated from BM as described in the Methods. DCs were cultured for 3 days with or without BM-MDSCs. The densities of major histocompatibility complex class II (MHC II) and CD86 maturation markers on the surface of DCs (CD11c+ cells) were determined by flow cytometry and the results expressed as mean fluorescence intensity (MFI). (A) Expression level of MHC II on the DCs (open bar) slightly increased in the presence of BM-MDSCs (closed bar), but this increase did not reach statistical significance (ns, not significant; p = 0.059; Mann-Whitney U test). (B) There was no significant difference in the expression level of CD86 on the DCs either when these cells were cultured without (open bar) and with (closed bar) BM-MDSCs (ns; p = 0.667; Mann-Whitney U test). Data shown are from 5 independent experiments. (TIF) [file pone.0111815.s003.tif]
