# Supplementary material for: Suppression of Proteoglycan-Induced Autoimmune Arthritis by Myeloid-Derived Suppressor Cells Generated In Vitro from Murine Bone Marrow
Source: PLoS One. 2014 Nov 4;9(11):e111815. doi: 10.1371/journal.pone.0111815 (PMC4219784; doi:10.1371/journal.pone.0111815)
Supplement: Table S1 — Concentrations of GM-CSF, IL-6, and G-CSF in synovial fluid (SF) and serum collected from arthritic (PGIA) mice. (DOCX) [file pone.0111815.s004.docx]

**Table S1. Concentrations of GM-CSF, IL-6, and G-CSF in synovial fluid (SF) and serum samples from arthritic (PGIA) mice^a^**

__________________________________________________________________

GM-CSF (ng/ml) IL-6 (ng/ml) G-CSF (ng/ml)

__________________________________________________________________

SF #1 201.79 3.71 5.16

SF #2 706.00 5.07 122.73

SF #3 654.77 3.54 71.38

SF #4 299.40 3.50 50.64

SF #5 200.39 1.91 10.67

__________________________________________________________________

SF Mean ± SEM 417.47 ± 111.13 3.54 ± 0.50 52.12 ± 21.54

__________________________________________________________________

Serum #1 11.76 0.63 5.78

Serum #2 28.80 1.42 7.58

Serum #3 8.61 0.54 3.72

Serum #4 7.07 0.44 4.79

Serum #5 12.31 0.39 5.22

__________________________________________________________________

Serum Mean ± SEM 13.71 ± 3.90 0.68 ± 0.19 5.41 ± 0.64

__________________________________________________________________

*P* value^b^ SF vs serum 0.008 0.003 0.056

__________________________________________________________________

^a^Each SF and serum sample was pooled from at least 3 mice.

^b^*P* value was calculated using the Student’s t test or the Mann-Whitney U test.
